# 11.F. Workshop: Can digital health literacy act as a protective factor for students in times of crisis?

**DOI:** 10.1093/eurpub/ckac129.709

**Published:** 2022-10-25

**Authors:** Organised by: University of Graz (Austria), EUPHA Working Group on Health Literacy, Chair persons: Barbara Gasteiger-Klicpera (Austria), Michaela Wright (Austria)

## Abstract

As mental health related issues become more and more prevalent across all ages and social groups around the globe, the identification of protective factors related to well-being is of high importance. In times of the COVID-19-pandemic, this proves to be even more crucial. Young adults and especially students were particularly burdened by social isolation and missing opportunities for personal exchange and supporting relationships during the pandemic. Within this workshop we will discuss the significance of a possible protective factor for the promotion and strengthening of well-being: digital health literacy (DHL). It involves the ability to search for health-related information, to add self-generated content, to evaluate the reliability of health information, to determine the relevance of health information and to protect one's privacy. Findings suggest that it plays a vital part as a protective resource when it comes to maintaining or promoting well-being. This might be particularly the case when the possibility of accessing health-related information is restricted due to reduced social contact. Since important sources for health information are available within the digital space, digital competencies are becoming important to access such information and to conduct a successful and healthy life. The competence of adequately dealing with digital health information, in particular, became more relevant during the COVID-19-pandemic. DHL can be seen as a two-dimensional construct. On the one hand, it refers to the ability to use digital resources to gather health information and, on the other hand, it refers to critical information literacy. Critical information literacy is the ability to collect, understand, evaluate and apply information. Within the proposed workshop, findings of a university survey, conducted within the global COVID-HL network, will be presented. Presentation 1 seeks to address the role of individual factors for the interaction between well-being and DHL. It will further examine the importance of being able to properly assess the relevance of health information. Presentation 2 sheds light on actions, such as adding self-generated health content, when it comes to mental health promotion. It will also take up the relevant individual factors that mediate the relationship between DHL and well-being. Presentation 3 highlights the ability of students to search for health-related information and to use it as a factor to improve their well-being. Presentation 4 provides insight into the importance of DHL for future health professionals in a health sector that is under digital transformation. Lastly, presentation 5 argues for the necessary enhancement of DHL and sense of coherence of students and stresses the need for health promoting and target group specific interventions. In a second step, the audience will be encouraged to ask questions and to engage in a discussion about the suggested conclusions and implications.

**Key messages:**

• It has been shown internationally that there is a strong relationship between digital health literacy and students’ well-being.

• Individual factors are relevant mediators in the relationship between well-being and digital health literacy.

